# The highly conserved region within exonuclease III-like in PML-I regulates the cytoplasmic localization of PML-NBs

**DOI:** 10.1016/j.jbc.2024.107872

**Published:** 2024-10-10

**Authors:** Xinxin Liang, Jinwen Chen, Peijie Yan, Zhongzhou Chen, Chao Gao, Rulan Bai, Jun Tang

**Affiliations:** 1 National Key Laboratory of Veterinary Public Health Security, Key Laboratory of Animal Epidemiology of the Ministry of Agriculture and Rural Affairs, College of Veterinary Medicine, China Agricultural University, Beijing, China; 2 State Key Laboratory of Animal Biotech Breeding, College of Biological Sciences, China Agricultural University, Beijing, China

**Keywords:** PML-I, exon 9, exon 8a, EXO-like-domain, subcellular localization

## Abstract

The sub-nuclear protein structure PML-NB regulates a wide range of important cellular functions, while its abnormal cytoplasmic localization may have pathological consequences. However, the nature of this aberrant localization remains poorly understood. In this study, we unveil that PML-I, the most conserved and abundant structural protein of PML-NB, possesses potent cytoplasmic targeting ability within the N-terminal half of the exonuclease III-like domain encoded by its unique exon 9, independent of the known nuclear localization signal. Fusion of this region to PML-VI can relocate PML-VI from the nucleus to the cytosol. Structural and deletion analysis revealed that the cytoplasmic targeting ability of this domain was restrained by the sequences encoded by exon 8a and the 3′ portion of exon 9 in PML-I. Deletion of either of these regions relocates PML-I to the cytosol. Furthermore, we observed a potential interaction between the ER-localized TREX1 and the cytoplasmic-located PML-I mutants. Our results suggest that perturbation of the EXO-like domain of PML-I may represent an important mode to translocate PMLs from the nucleus to the cytosol, thereby interfering with the normal nuclear functions of PML-NBs.

Promyelocytic leukemia protein (PML) nuclear bodies (NBs), also known as ND10 (1), PODs (2), *etc.*, are dynamic membrane-less nuclear substructures commonly found in mammalian cells. They are highly dynamic multiprotein complexes that serve as platforms for nuclear biochemical reactions and regulate a variety of functions, including apoptosis and cellular senescence (3), inhibition of proliferation (4), maintenance of genome stability (5) and antiviral responses (6, 7, 8, 9). Defects in PML-NBs are closely associated with pathogenesis, particularly tumorigenesis (10).

PML serves as the organizer of the NBs (11). The PML gene contains nine exons (12). Exons 1 to 3 encode an RBCC region, encompassing the RING finger, B1 and B2 boxes, and a Coiled-Coil motif (13). Exons 4 to 9 are subject to alternative splicing, producing various C-terminally distinct isoforms that are associated with specific cellular functions and protein interactions (14, 15). PML-I, the largest isoform, is a highly expressed and conserved isoform, potentially playing a crucial role in PML function (15, 16, 17). Like other nuclear isoforms, PML-I displays strong nuclear localization due to the presence of a nuclear location signal (NLS) on exon 6 (16). However, unlike others, PML-I harbors a functional nuclear export signal (NES) on its unique exon 9, capable of shuttling PML-I protein between the nucleus and the cytoplasm (18, 19). The C-terminus of PML-I also contains a highly conserved EXO III-like structural domain, which shares homology with the DEDDh family of nucleic acid exonucleases (19, 20).

Despite its predominant nuclear localization, cytoplasmic localization of PML has been reported, especially in certain cancers. For instance, significant cytoplasmic staining for PML proteins was observed in the biopsy of a patient with skin cancer, accompanied by a small number of NBs in the nucleus (17). Cytoplasmic localization of PML has also been observed in a large number of hepatocellular carcinomas (21, 22). Aberrant cytoplasmic localization of PML is potentially linked to specific mutations or pathological conditions. In some cases, the cytoplasmic distribution of PML is attributed to the absence of NLS in PML due to alternative splicing or mutations, as in the case of acute promyelocytic leukemia (23, 24, 25, 26, 27, 28). However, in the majority of cases, the nature of this aberrant localization remains poorly understood. Interestingly, a murine PML isolated from a murine plasmacytoma cell line displays cytoplasmic localization despite containing an NLS (29), indicating that other mechanisms are involved.

Like nuclear PMLs, cytoplasmic PMLs also regulate a wide range of cellular functions, including the TGF-β signaling pathway (30, 31), p53 pathway (32), metabolism (33) and apoptosis (34, 35). However, aberrant cytoplasmic localization of PML may inappropriately activate the cytoplasmic functions of PML while impairing its normal nuclear functions, contributing to pathogenesis.

In this study, we identified that the EXO-like domain within the amino acid sequence encoded by the 5′ half of exon 9 in PML-I contains a region capable of targeting PML-I to the cytosol independent of the NES. For simplicity, the amino acid sequence encoded by an exon is also referred to as the exon. Furthermore, we found the cytoplasmic targeting ability of this region is inhibited by exon 8a and the C-terminal half of exon 9 in the normal context of PML-I. Our results suggest that any structural perturbation resulting in the exposure of this region of PML-I may alter the subcellular localization of PML, thereby, regulating PML-NB mediated cell functions.

## Results

### Deletion of exon 8a in PML-I relocates the PML protein from the nucleus to the cytoplasm

**Figure 1:**
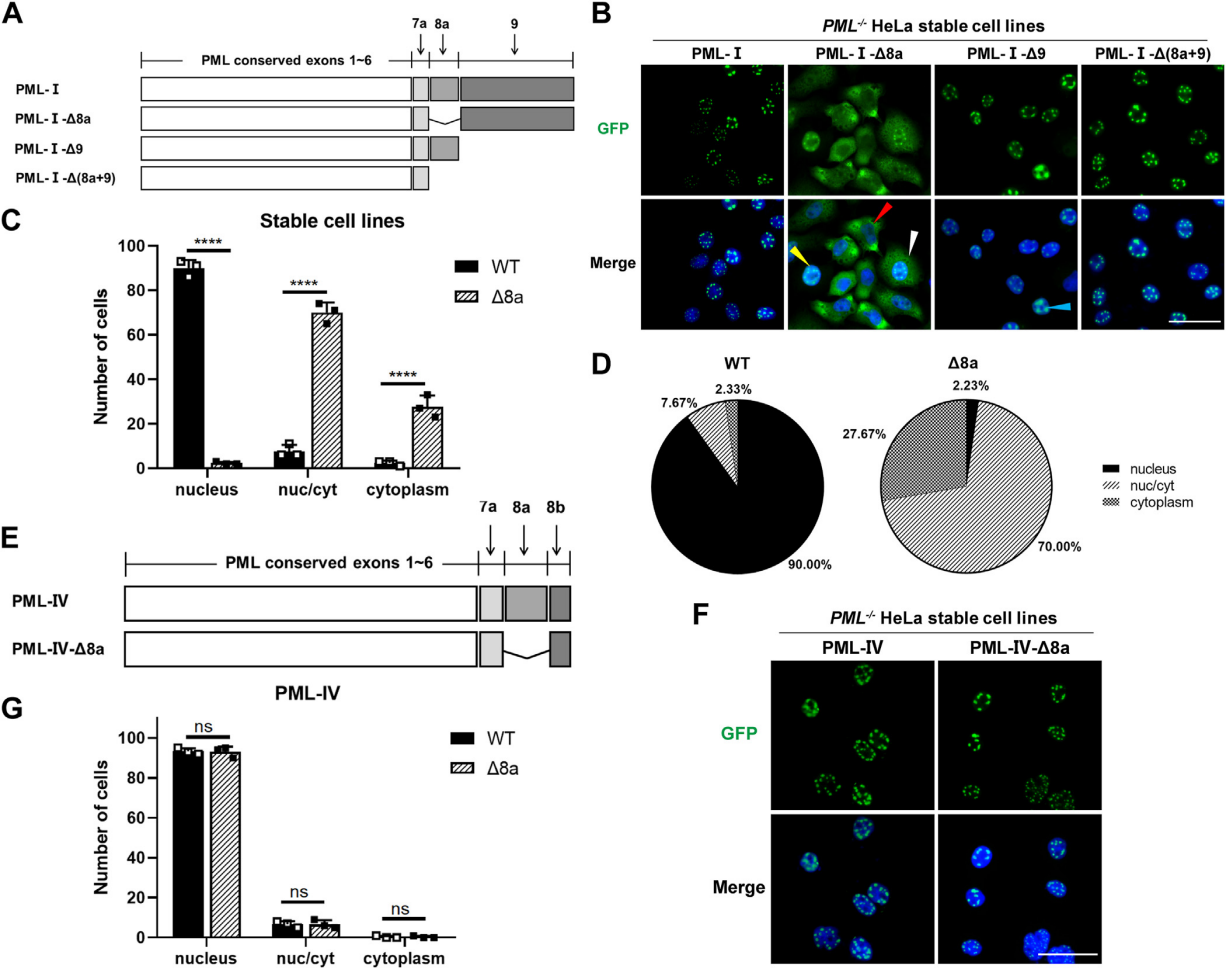
**Deletion of exon 8a leads to PML-I cytoplasmic localization.***A*, schematic illustration of human PML-I and its deletion mutants, including PML-I-Δ8a, -Δ9 and -Δ(8a+9), where exon 8a (aa 571-620), exon 9 (aa 621-882), or both were deleted, respectively. *B*, fluorescence microscopic analysis of the localization of PML-I and its deletion mutants. Stable cell lines expressing GFP-tagged PML-I, -Δ8a, -Δ9 and -Δ(8a+9) were generated in PML-KO HeLa cells and then analyzed by fluorescence microscopy. Nuclei were stained with DAPI. PML-I-Δ8a exhibits three localization patterns: primarily cytoplasmic (*red arrow*), primarily nuclear (*yellow arrow*), and both cytoplasmic and nuclear (*white arrow*). Scale bar: 50 μm. *C* and *D*, the relative proportion of each pattern in PML-I-Δ8a expressing was calculated and presented by averaging three independent counts of 100 cells from randomly chosen fields, compared to that of PML-I wild-type cells. *E*, schematic depiction of PML-IV and its 8a deletion mutant (PML-IV-Δ8a). *F*, stable cell lines expressing GFP-tagged PML-IV and PML-IV-Δ8a were generated in PML-KO HeLa cells and then analyzed by fluorescence microscopy. Nuclei were stained with DAPI. Scale bar: 50 μm. *G*, the average number of each pattern in PML-IV-Δ8a expressing was calculated and presented by averaging three independent counts of 100 cells from randomly chosen fields, compared to that of PML-IV wild-type cells. Bars and error bars in (*C* and *G*) represent the mean with s.d. (n = 3). ∗∗∗∗*p* < 0.0001; ns, not significant.

PML-I is the most conserved and abundant isoform, potentially influencing the overall localization of PML-NBs (17). We therefore considered the possibility that the reported cytoplasmic localization of PML-NBs may relate to PML-I. Compared to other nuclear PML isoforms, PML-I is unique in possessing exon 9. It also shares exon 8a with PML-IV. To determine whether PML-I has a propensity to localize in the cytoplasm, we generated two PML-I deletion mutants, PML-I-Δ8a and PML-I-Δ9, and fused each with a GFP N-terminally (Fig. 1*A*). The cellular localization of these mutants was examined in the PML-knockout (PML-KO) HeLa cell line. Interestingly, expression of PML-I-Δ8a in PML-KO HeLa cells exhibited significant cytoplasmic localization, contrasting with the classical nuclear localization of PML-I, while PML-I-Δ9 was still predominantly nuclear, exhibiting enlarged NBs. To confirm the cytoplasmic localization of PML-I-Δ8a, we generated stable cell lines expressing GFP-tagged PML-I or PML-I-Δ8a (Fig. 1*B*). Compared to PML-I, mainly located in the nucleus (about 90%), the majority of PML-I-Δ8a (about 97.67%) exhibited cytoplasmic distribution, including 70.00% in both nucleus and cytosol and 27.67% prominently cytoplasmic, with only 2.23% exhibiting typical NBs (Fig. 1, *C* and *D*). These results indicate that the deletion of exon 8a causes the translocation of PML-I from the nucleus to the cytoplasm.

PML-IV contains exon 8a and shares exon 1 to 8a with PML-I (14). We therefore examined the impact of deleting exon 8a on the localization of PML-IV. PML-IV exon 8a deletion mutant (PML-IV-Δ8a) was constructed and fused with GFP (Fig. 1*E*). Corresponding stable cell lines were generated. GFP fluorescence observation revealed no discernible differences in the localization between PML-IV and PML-IV-Δ8a. Both showed a typical NB phenotype (Fig. 1, *F* and *G*).

The observation that deletion of 8a causes cytoplasmic localization only in PML-I, but not in PML-IV suggests that the unique exon 9 of PML-I may be responsible for the abnormal cytoplasmic localization of PML-I-Δ8a. To confirm this, we further removed exon 9 from PML-I-Δ8a and constructed a GFP-PML-I-Δ(8a+9) mutant (Fig. 1*A*). Both transient expression and stable cell lines showed that PML-I-Δ(8a+9) restored the typical NBs phenotypes (Fig. 1*B*). Together, these data suggest that exon 9 contains the information that directs PML-I-Δ8a to the cytosol.

### Cytoplasmic localization of PML-I induced by exon 8a deletion leads to loss of antiviral function in PML-NBs

**Figure 2:**
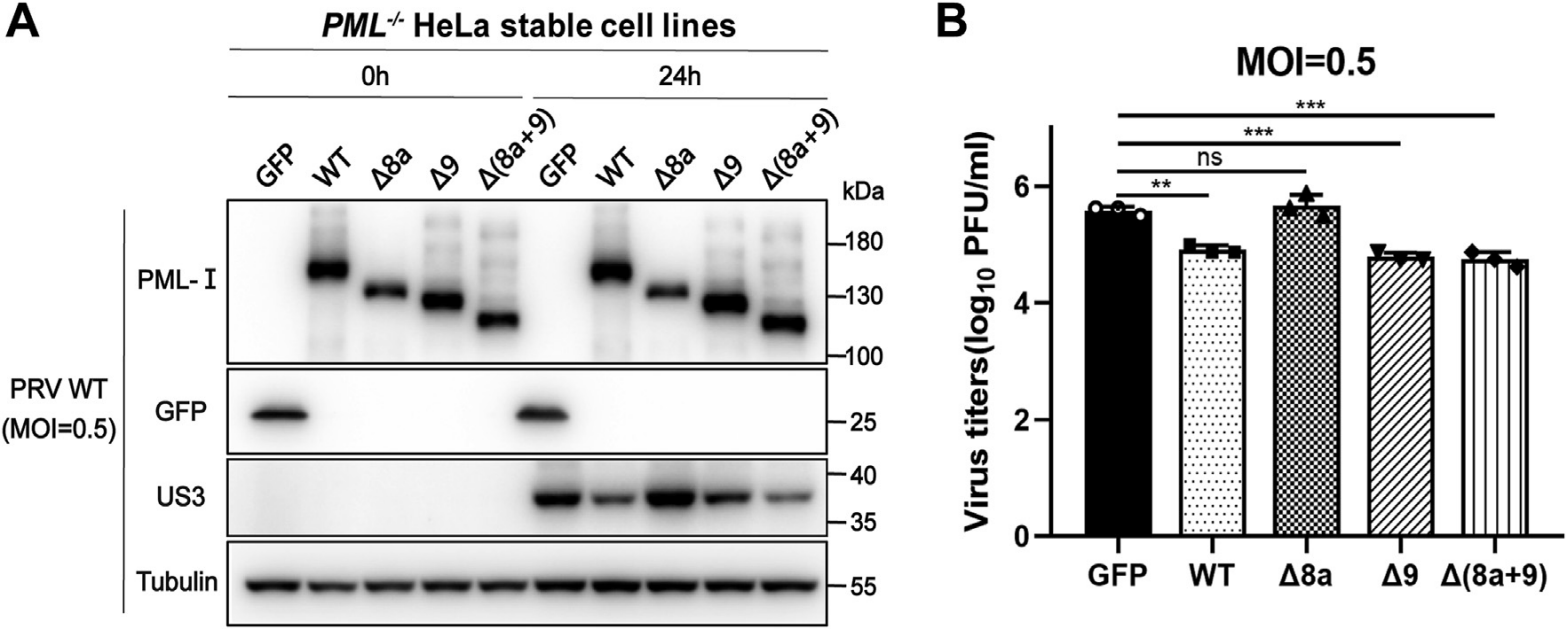
**Cytoplasmic localization of PML-I due to exon 8a deletion disrupts the antiviral function of PML-NBs.***A* and *B*, stable cell lines expressing PML-I and its mutants [Δ8a, Δ9, Δ(8a+9)] were infected with PRV at 0.5 MOI. Cell samples were collected after 24 h for western blotting, while supernatants were used for viral titer assay. Bars and error bars in (*B*) represent the mean with s.d. (n = 3). ∗∗*p* < 0.01; ∗∗∗*p* < 0.001; ns, not significant.

To examine whether the cytoplasmic localization of PML-I-Δ8a affects the nuclear functions of PML-NBs, we infected the aforementioned stable cell lines with a model alpha-herpesvirus, Pseudorabies virus (PRV). Western blotting analysis of the expression of the viral protein US3 and viral titer measurement showed that PML-I and its nuclear mutants, PML-I-Δ9 and PML-I-Δ(8a+9), exhibited significant anti-viral activity compared to the control. In contrast, cytoplasmic PML-I-Δ8a lost this ability (Fig. 2, *A* and *B*). These results suggest that the change in PML localization impacts its function.

SUMOylation of PML is critically involved in PML-NB formation (36). Western blotting showed that PML-I, -Δ9, and -Δ(8a+9) exhibited multiple bands indicative of SUMOylation. However, interestingly, PML-I-Δ8a showed only one band (Fig. 2*A*), suggesting it is not sufficiently SUMOylated. To confirm this, SUMOylation assays were performed by co-expressing Flag-PML-I proteins with HA-SUMO-1 in PML-KO HeLa cells, followed by immunoprecipitation with a Flag antibody. Western blotting with a HA antibody revealed that while WT PML-I is SUMOylated, PML-I-Δ8a lacks SUMOylation (Fig. S1). It would be interesting to determine the mechanism involved in the future.

### Exon 9 in PML-I mediates the cytoplasmic location of PML mutant proteins independently of its NES

**Figure 3:**
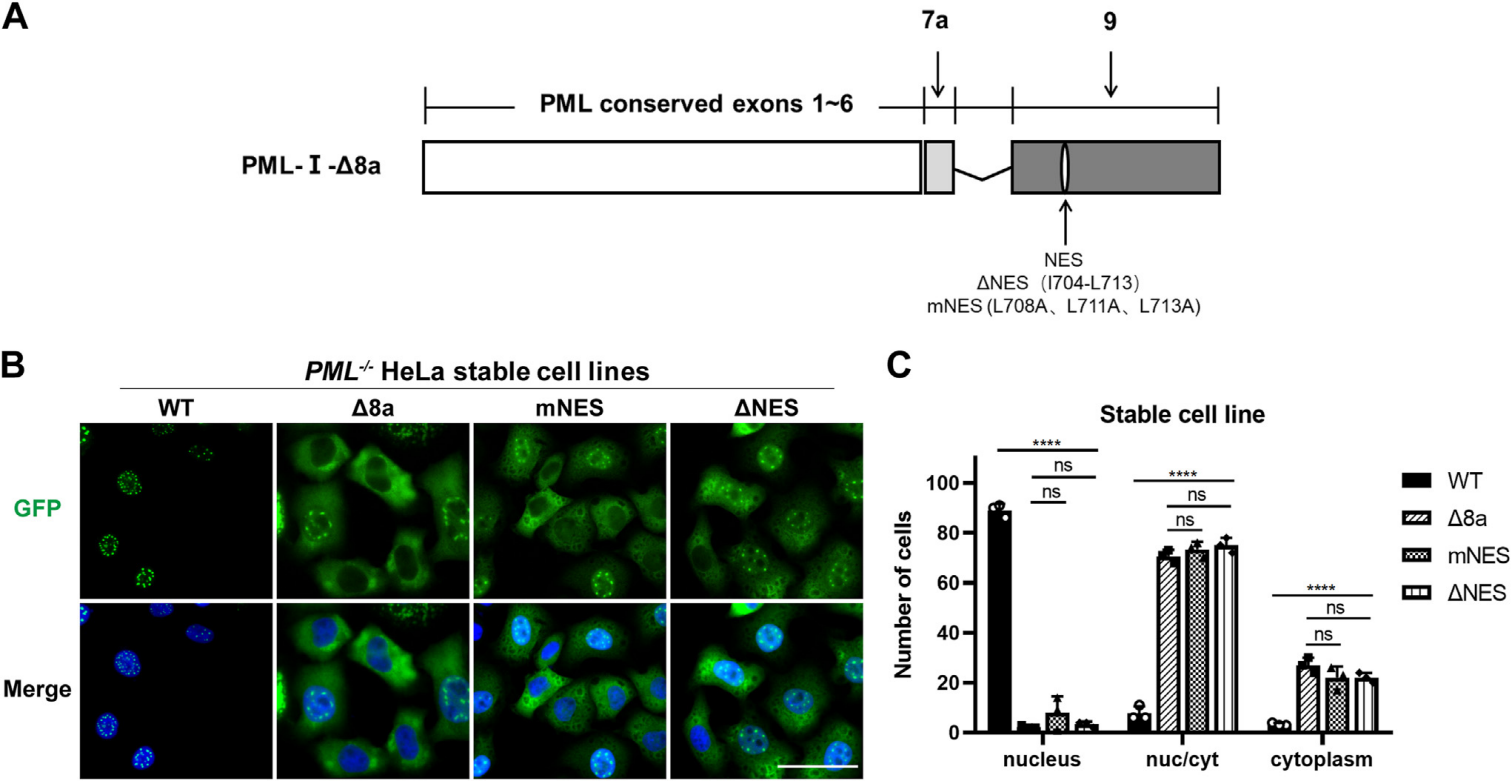
**Deletion of exon 8a induced cytoplasmic localization of PML-I is independent of the NES.***A*, schematic representation of deletion and point mutations of NES in GFP-PML-I-Δ8a. *B*, stable cell lines expressing GFP-PML-I-mNES/-ΔNES were generated in PML-KO HeLa cells and then analyzed by fluorescence microscopy. Nuclei were stained with DAPI. Scale bar: 50 μm. *C*, the average number of each localization patterns in 100 cells of each stable cell line in (*B*) was calculated and shown. Bars and error bars in (*C*) represent the mean with s.d. (n = 3). ∗∗∗∗*p* < 0.0001; ns, not significant.

Since exon 9 contains a nuclear export signal (NES) at the aa 704 to 713 (37), we wondered whether the deletion of 8a in PML-I activates the NES, thereby mediating its cytoplasmic localization. To test this, we either mutated (mNES) or completely deleted (ΔNES) the NES and generated stable cell lines expressing GFP-PML-I-Δ8a-mNES or –ΔNES (Fig. 3*A*). However, both mutants exhibited a similar localization pattern to PML-I-Δ8a, mostly distributed in the cytoplasm, with NBs appearing in some nuclei and none in others (Fig. 3, *B* and *C*). These results suggest that the cytoplasmic location of PML-I-Δ8a may depend on another region in exon 9 independent of the NES.

### The EXO-like domain within exon 9 in PML-I possesses a distinct cytoplasmic targeting ability

**Figure 4:**
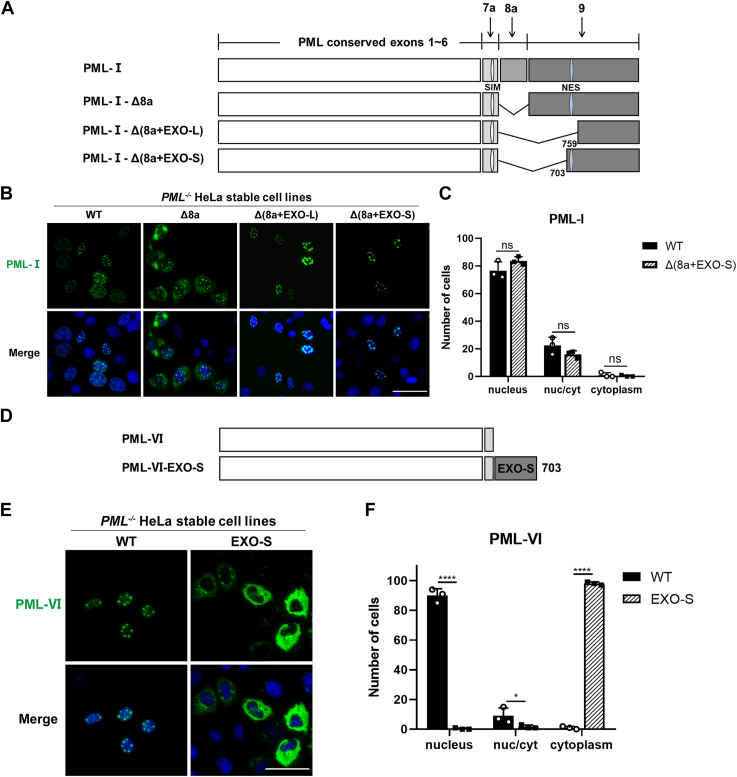
**The EXO-S region in exon 9 of PML-I mediates cytoplasmic localization of PML proteins.***A*, schematic diagram of the PML-I deletion mutants. Exon 8a and EXO-L domain were deleted in PML-I-Δ(8a+EXO-L). Exon 8a and EXO-S domain were deleted in PML-I-Δ(8a+EXO-S). *B*, GFP fluorescence analysis of PML-I mutants’ localization. PML-KO HeLa cells transfected with PML-I-Δ(8a+EXO-L) and -Δ(8a+EXO-S) mutants were fixed after 24 h and analyzed by GFP fluorescence. Scale bar: 50 μm. *C*, the average number of each phenotype in 100 cells for each transfection in (*B*) was calculated and shown. *D*, schematic representation of PML-VI-EXO-S. EXO-S domain (621-703aa) was added to the C-terminal of PML-VI. *E*, GFP fluorescence analysis of the localization of PML-VI-EXO-S transiently expressed in PML-KO HeLa cells. *F*, the average number of each phenotype in 100 cells for each transfection in (*E*) was calculated and shown. Scale bar: 50 μm. Bars and error bars in (*C* and *F*) represent the mean with s.d. (n = 3). ∗*p* < 0.05; ∗∗∗∗*p* < 0.0001; ns, not significant.

To identify the sequence in exon 9 responsible for mediating the cytoplasmic localization of PML-I-Δ8a, we generated a series of GFP-tagged deletion mutants based on PML-I-Δ8a and examined their localizations in PML-KO HeLa cells. Deletion of aa 621 to 759, which encodes a majority portion of a highly conserved nucleic acid exonuclease type III (EXO-III)-like structural domain (20) and is designated as PML-I-Δ(8a+EXO-L), restored the nuclear localization of PML generating normal NBs. We further shortened the deletion region to 621-703aa, excluding the NES, and the mutant was still able to form normal NBs in the nucleus, which we named as the EXO-S structural domain (Fig. 4, *A*–*C*). These results suggest that the EXO-S domain may mediate the cytoplasmic localization of PML-I-Δ8a.

To further confirm the cytoplasmic targeting ability of the EXO-S region, we fused this region to the C-terminus of PML-VI (Fig. 4*D*). PML-VI is the shortest nuclear PML isoform, with the C-terminus encoded only by exons 4, 5, 6, and part of 7a (14, 16). Strikingly, the fusion of the EXO-S domain altered the localization of PML-VI from the nucleus to the cytoplasm (Fig. 4, *E* and *F*).

Collectively, these results suggest that in addition to NES, the EXO-S domain located at the N-terminus of exon 9 also contributes to the cytoplasmic localization of PML proteins.

### The cytoplasmic targeting ability of EXO-like is inhibited by both exon 8a and the C-terminal region of exon 9

**Figure 5:**
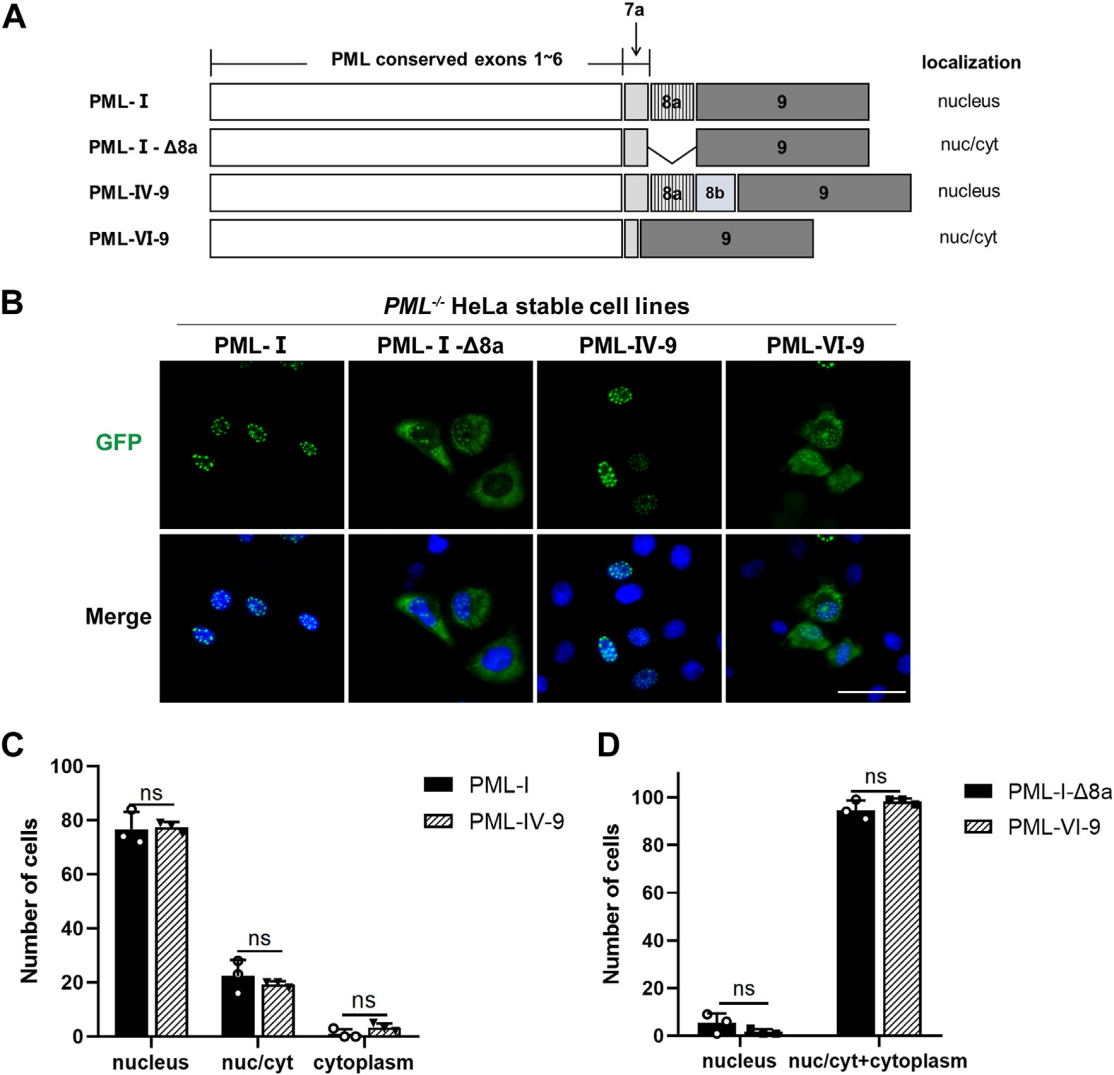
**Exon 8a inhibits exon 9-mediated cytoplasmic localization of PML proteins.***A*, schematic representation of the mutants formed by fusion of the C-terminus of PML-IV and PML-VI with exon 9. *B*, GFP fluorescence was used to observe the subcellular localization of each mutant in (*A*). Scale bar: 50 μm. *C* and *D*, the average number of each phenotype in 100 cells for each transfection in (*B*) was calculated and shown. Bars and error bars in (*C* and *D*) represent the mean with s.d. (n = 3). ns, not significant.

Although the EXO-S domain can translocate PML-IV and PML-I-Δ8a from the nucleus to the cytoplasm, this ability is inhibited in the presence of exon 8 in PML-I. This suggests that exon 8a may limit the cytoplasmic targeting ability of EXO-S. Thus, we suspected that fusing exon 9 to the C-terminus of PML-IV (PML-IV-9) might not be able to translocate PML-IV to the cytoplasm, as it also contains exon 8a. Indeed, PML-IV-9 still exhibited nuclear localization despite the cytoplasmic localization of PML-VI-9 (Fig. 5, *A* and *B*). Cell counting of different phenotypes also indicated that PML-IV-9 resembled PML-I, mainly localized in the nucleus, whereas PML-VI-9 resembled PML-I-Δ8a, primarily localized in the cytoplasm (Fig. 5, *C* and *D*). The results further confirm that the presence of exon 8a represses the EXO-S in exon 9.

**Figure 6:**
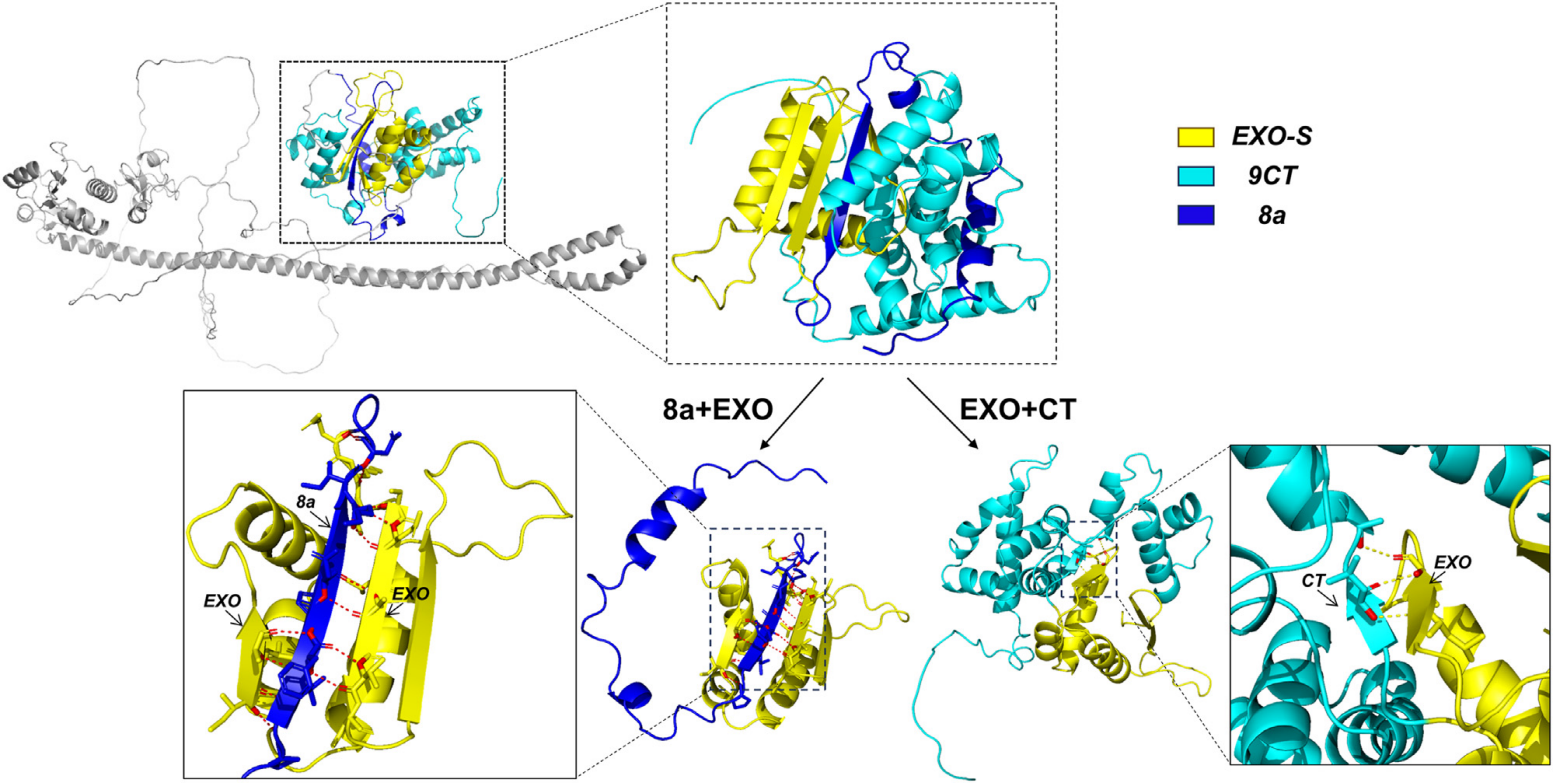
**Analysis of exon 8a, EXO-S domain, and exon 9 C-terminus interactions based on protein 3D structure predictions.** The three-dimensional structure of human PML-I was obtained from the AlphaFold database and visualized using PyMOL software. *Dark blue* marks exon 8a, *yellow* marks the EXO-S structural domain, and *light blue* marks the C-terminus of exon 9.

To better understand the inhibitory effect of exon 8a on EXO-S structurally, we obtained the predicted three-dimensional structure of the human PML-I protein from the AlphaFold database (https://AlphaFold.ebi.ac.uk/entry/P29590) (38) and analyzed the relationship between exon 8a and EXO-S using PyMOL (39). Our analysis revealed that exon 8a, particularly its C-terminal half, directly interacts with the EXO-S region in exon 9 (Fig. 6). We speculate that this interaction may impede the cytoplasmic targeting function of the EXO-S region, thereby promoting the nuclear localization of PML-I.

**Figure 7:**
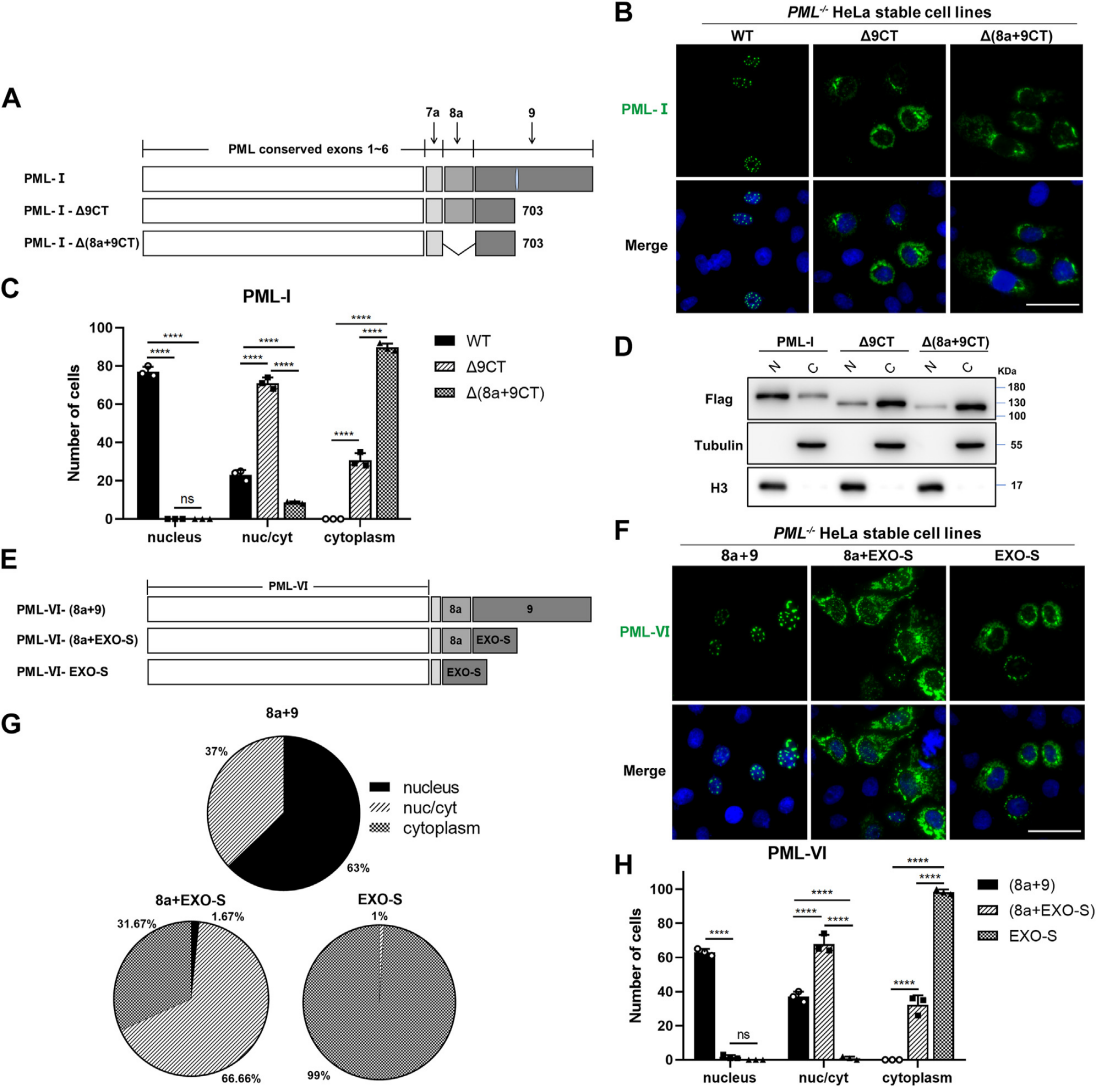
**C-terminal of exon 9 inhibits EXO-mediated cytoplasmic localization of PML proteins.***A*, schematic representation of PML-I-Δ9CT and PML-I-Δ(8a+9CT), where the C-terminal of exon 9 alone or together with exon 8a was deleted, respectively. *B* and *C*, subcellular localization of PML-I-Δ9CT and PML-I-Δ(8a+9CT) was analyzed by GFP fluorescence and phenotype counting. Scale bar: 50 μm. *D*, flag-tagged PML-I, PML-I-Δ9CT or PML-I-Δ(8a+9CT) was transfected into PML-KO HeLa cells. The nuclear and cytoplasmic distribution of PML proteins was analyzed using a nuclear-cytoplasmic fractionation assay. Flag indicates PML proteins, tubulin serves as a cytoplasmic control, and H3 serves as a nuclear control. “N” represents the nucleus and “C” represents the cytoplasm. *E*, schematic diagram of the PML-VI fusion proteins. PML-VI was C-terminally fused with exon 8a and 9 of PML-I [PML-VI-(8a+9)], exon 8a plus EXO-S [PML-VI-(8a+EXO-S)], or EXO-S alone (PML-VI-EXO-S). *F*–*H*, subcellular localization analysis of PML-VI fusion proteins. PML-KO HeLa cells were transfected with plasmids as indicated in (*E*) and analyzed by GFP fluorescence (*F*). Scale bar: 50 μm. The average number and proportion of each phenotype for each fusion protein in 100 cells were calculated and shown (*G* and *H*). Bars and error bars in (*C* and *H*) represent the mean with s.d. (n = 3). ∗∗∗∗*p* < 0.0001; ns, not significant.

The structural analysis also revealed the C-terminal region of exon 9 in PML-I (9CT) interacting with EXO-S. We suspected that this interaction might also interfere with the cytoplasmic targeting ability of EXO-S (Fig. 6). To test this, we deleted this region (aa704-882) from PML-I, generating the mutant PML-I-Δ9CT (Fig. 7*A*). Fluorescent microscopy showed that PML-I-Δ9CT exhibited a significantly different localization pattern compared to PML-I. Despite the presence of NBs, it showed considerable cytoplasmic localization (Fig. 7, *B* and *C*). We performed a nuclear-cytoplasmic fractionation assay to validate the results shown in Figure 7*B*. Consistent with our imaging data, the fractionation assay also demonstrated that PML-I was predominantly present in the nucleus, while both PML-I-Δ9CT and PML-I-Δ(8a+9CT) were mainly distributed in the cytoplasm, with PML-I-Δ(8a+9CT) showing a more pronounced trend towards cytoplasmic localization (Fig. 7*D*). These result indicates that the C-terminal region of exon 9 also plays a role in restraining EXO-S and that 8a alone is insufficient to fully inhibit it.

To further confirm this, we fused exon 8a and EXO-S with or without 9CT to PML-VI, generating mutants PML-VI-(8a+9), which is 8a+EXO-S+9CT, and PML-VI-(8a+EXO-S) (Fig. 7*E*), and compared their localization with PML-VI-EXO-S. Fluorescent microscopy showed that PML-VI-EXO-S exhibited the highest degree of cytoplasmic localization, followed by PML-VI-(8a+EXO-S), while PML-VI-(8a+9) is mostly in the nucleus (Fig. 7, *F*–*H*).

Taken together, these data indicate that EXO alone can translocate PML from the nucleus to the cytoplasm, but this ability of EXO is inhibited in PML-I by 8a and 9CT.

### PML-I cytoplasmic mutants relocalize different nuclear PML isoforms from the nucleus to the cytoplasm

**Figure 8:**
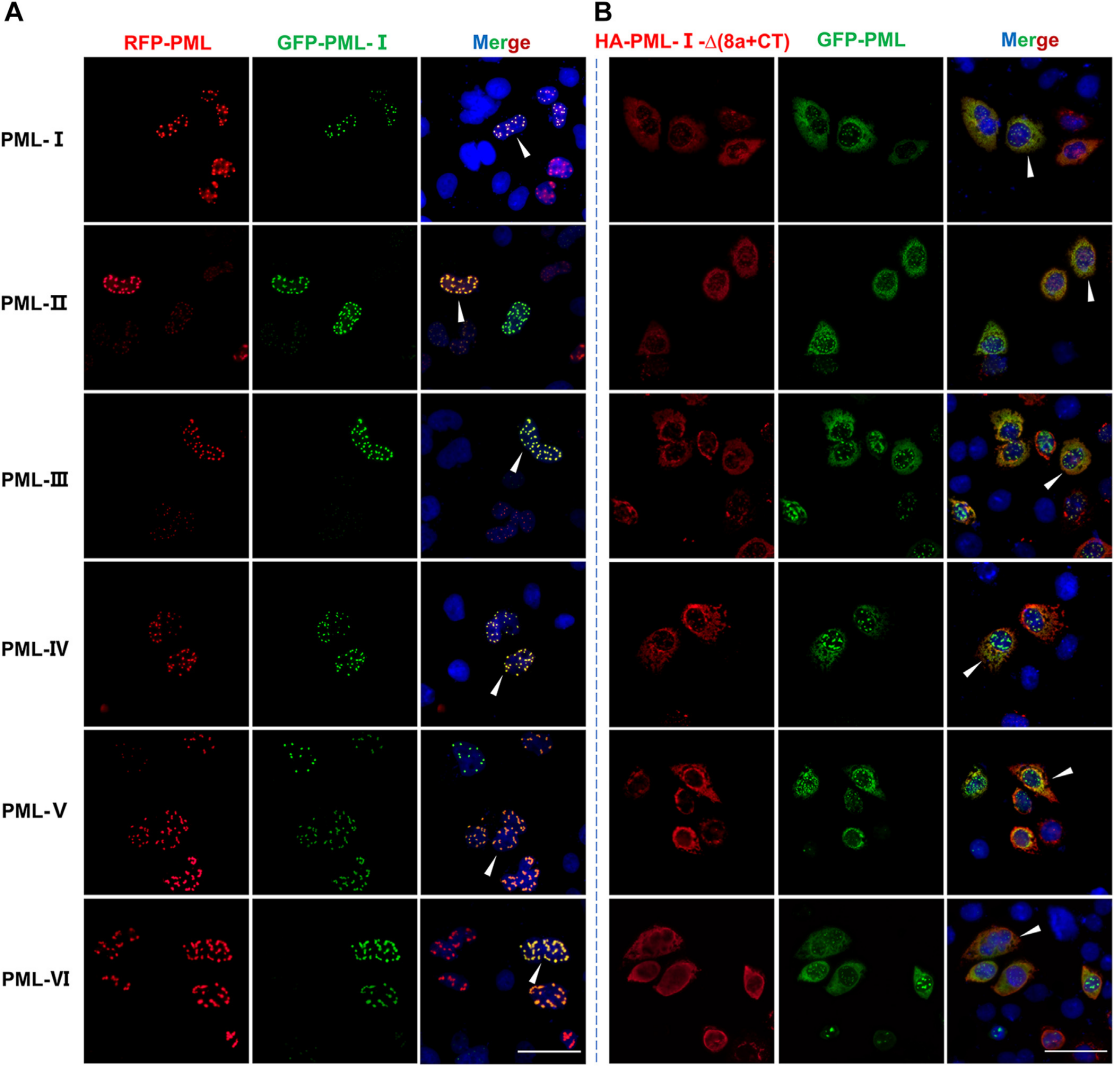
**PML-I mutant alters the subcellular localization of other nuclear PML isoforms.***A*, RFP-PML-I∼VI was co-transfected with GFP-PML-I into PML-KO HeLa cells and the subcellular localization of each PML isoform was observed by fluorescence microscopy. After 24 h, the cells were fixed. *B*, GFP-PML-I∼VI was co-transfected with HA-PML-I-Δ(8a+9CT) into PML-KO HeLa cells. After 24 h, the cells were fixed and then immune-stained with anti-HA antibodies, followed by TRITC-labeled secondary antibodies. The subcellular localization of each PML subtype was observed by fluorescence microscopy. *White arrows* indicate co-localization of intracellular PML-I, PML-I-Δ(8a+9CT) with different PML isoforms. Scale bar: 50 μm.

Since different PML isoforms can heterodimerize *via* their RBCC domain, we hypothesized that the cytoplasmic distribution of PML-I mutants may affect the localization of other isoforms (14). To test this, we co-transfected various PML isoforms (PML-I∼VI) with either PML-I or PML-I-Δ(8a+9CT) into PML-KO HeLa cells. Fluorescence microscopy showed PML-I and PML-I∼VI form PML-NBs in the nucleus (Fig. 8*A*), while PML-I∼VI co-localizes with PML-I-Δ(8a+9CT) in the cytoplasm (Fig. 8*B*). These findings suggest that the cytoplasmic localization of PML-I promotes the cytoplasmic redistribution of other isoforms, potentially impairing the formation and nuclear functions of PML-NBs.

### Cytoplasmic PML-I mutants co-localize and interact with TREX1

**Figure 9:**
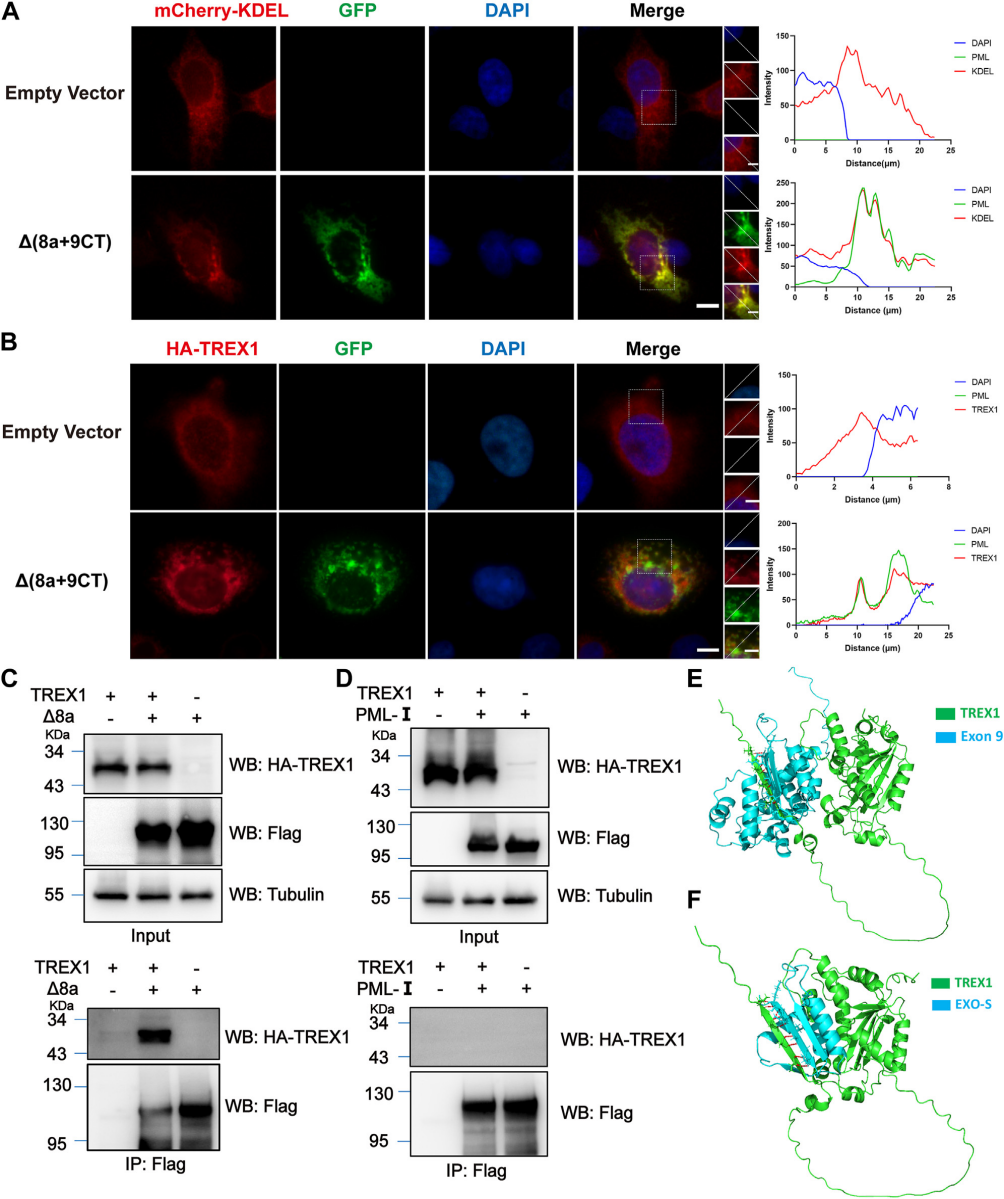
**TREX1 interacts and co-localizes with cytoplasmic PML mutants.***A* and *B*, immunofluorescence analysis of co-localization of Flag-PML-I-Δ(8a+9CT) with the ER or with TREX1. GFP-PML-I-Δ(8a+9CT) was co-expressed with mCherry-KDEL (*A*) or HA-TREX1 (*B*) in PML-KO HeLa cells. After 24 h, the cells were fixed. The cells were then immune-stained with anti-HA antibodies, followed by TRITC-labeled secondary antibodies (*B*). Fluorescence microscopy was conducted to examine the co-localization of PML mutants with KDEL or TREX1. The line plots for the region of interest are presented at the *right*. Scale bar on the *left* images: 10 μm; scale bar on the *right zoom panels*: 5 μm. *C* and *D*, comparative analysis of interaction between TREX1 and PML-I or -Δ8a. HEK293T cells were co-transfected with HA-TREX1 and Flag-PML-WT or Flag-PML-I-Δ8a mutant. Cell lysates were immunoprecipitated and then analyzed by western blotting using anti-HA and anti-Flag antibodies. *E*, protein structures were predicted with AlphaFold (https://AlphaFold.ebi.ac.uk/) and molecular docking was performed. PyMOL was used for visualization. Exon 9 and the EXO-S domain may interact with TREX1. *Green* represents TREX1 and *blue* represents the PML section.

Next, we explored how the PML-I mutants are targeted to the cytoplasm. We hypothesized that the EXO-S domain of PML-I might interact with certain cytoplasmic structures or proteins thereby retaining PML proteins in the cytoplasm. Previous studies have shown that the C-terminus of PML-I, particularly the EXO-III region, shares sequence similarity with the DEDDh exonuclease TREX1 in mammals (19). Additionally, it has been shown that TREX1 typically forms homodimers at the endoplasmic reticulum (ER) Biochemical properties of mammalian TREX1 and its association with DNA replication and inherited inflammatory disease (40, 41, 42). Since extranuclear PML has also been reported to localize at ER in cells (34), we considered the possibility that EXO-S interacts with TREX1, thereby retaining PML-I-Δ8a in the cytoplasm, particularly at the ER. To test this hypothesis, the cytoplasmic PML mutant PML-I-Δ(8a+9CT) was co-expressed with either an ER-marker, mCherry-tagged KDEL, or with HA-TREX1 in PML-KO HeLa cells. Fluorescence microscopy was then performed to observe the co-localization of the PML mutants with KDEL or TREX1. Fluorescent microscopic analysis revealed that PML-I-Δ(8a+9CT) co-localized with mCherry-tagged KDEL and with TREX1, respectively (Fig. 9, *A* and *B*). Moreover, Flag-PML-I and Flag-PML-I-Δ8a were also individually co-expressed with HA-TREX1 in HEK293T cells. Co-immunoprecipitation assay demonstrated that Flag-PML-I-Δ8a interacted with HA-TREX1, whereas Flag-PML-I did not (Fig. 9, *C* and *D*). Taken together, these results suggest that the cytoplasmic PML-I mutants interact with TREX1 at the ER.

Moreover, we structurally analyzed the potential complex formation between TREX1 and PML mutants using the AlphaFold database (38) and PyMOL (39). Our analysis revealed that the deletion of exon 8a may result in the interaction of the C-terminus of PML-I with TREX1 (Fig. 9, *E* and *F*).

## Discussion

In this study, we unveil that the vestigial exonuclease domain of PML-I contains a potent cytoplasmic retention element that is effectively repressed in the normal PML-I context. Our findings highlight the crucial need to control the structure of the EXO-III and offer new insights into the cytoplasmic localization of nuclear PML, which could significantly impact its functions.

The C-terminal region of human PML-I encompasses a highly conserved putative exonuclease-III-like motif spanning residues 610 to 759 (20). Although human PML-I is primarily nuclear and lacks exonuclease activity, its ancient counterpart, spotted gar PML (sgPML), exhibits cytoplasmic localization along with exonuclease activity (19). The finding that the region of 621-703aa (EXO-S) within the N-terminal half of exon 9 of human PML-I can shift the localization of nuclear PML to the cytoplasm supports the cytoplasmic origin of PML-I (19). One possible explanation for the differential localization between human PML-I and spotted gar PML is that EXO-S is constrained by both exon 8a and the C-terminal half of exon 9 in human PML-I, while this restriction is likely weaker in sgPML. Analysis of sgPML using the reported program (43) reveals that it contains a putative NLS (376-SVKRAREDDQETEWIDKHRNQ-396), suggesting sgPML can localize in the nucleus. However, compared to human PML-I, the C-terminal region of sgPML lacks partial sequences at the C-terminal ends of both exon 8a (596-607aa) and exon 9 (856-882aa) (Fig. S2). The absence of sequences in both regions in sgPML could impact their interactions with EXO-S, leading to the cytoplasmic targeting of sgPML. This hypothesis is supported by the observation that sg8a does not inhibit EXO-S in the same way as human 8a (Fig. S3).

The identification of EXO-S as a cytoplasmic retention element suggests a novel mechanism that could alter the localization of PML proteins from the nucleus to the cytoplasm. PML-I is the predominant PML isoform in cells. Its localization is believed to dictate the overall localization of PML-NBs, as PML proteins heterodimerize *via* their RBCC regions (14, 44). Unlike other isoforms, PML-I possesses both an NLS and an NES, enabling it to shuttle between the nucleus and the cytoplasm. Nuclear export mediated by the NES is CRM1-dependent and highly regulated (18). Under normal conditions, PML-I is predominantly nuclear with its cytoplasmic output increasing under certain stresses in a CRM1-dependent manner (19, 37). In contrast, the cytoplasmic localization of PML mediated by EXO-S occurs in the absence of the NES and without any stress stimulation. One possible mechanism for EXO-S targeting PML in the cytoplasm involves its interactions with other cytoplasmic proteins. Intriguingly, we observed an interaction between PML-EXO-S and TREX1. As a member of the EXO-III family, TREX1 acts as a functional counterpart to the exonuclease of sgPML in mammals, responsible for clearing cytoplasmic DNAs. Further investigation is necessary to assess the impact of this interaction on the function of TREX1.

EXO-S is constrained by both exon 8a and the C-terminal half of exon 9 in PML-I, suggesting that if its potent cytoplasmic retention capability is not tightly regulated, it could compromise the normal functions of PML-NBs in the nucleus and/or lead to abnormal augmentation of cytoplasmic PML activities, potentially contributing to pathogenesis. Aberrant cytoplasmic localization of PML has been noted in viral infections and cancer. For instance, examination of skin cancer biopsies reveals a high cytoplasmic expression of PML proteins (17). Analysis of several clinical databases (GDC database, https://portal.gdc.cancer.gov/; Cosmic database, https://cancer.sanger.ac.uk/cosmic; cBioPortal database, https://www.cbioportal.org/; Clinvar database, https://www.ncbi.nlm.nih.gov/clinvar/) has revealed frequent occurrence of exon 9 C-terminal mutations in samples of various cancer types including malignant melanoma (45), intestinal adenocarcinoma (46), stomach adenocarcinoma, cutaneous melanoma, hepatocellular carcinoma (32), gastric cancer, intestinal adenocarcinoma, and endometrioid carcinoma (Fig. S4). These mutations can result in mutant PML-I proteins with partial deletions at the C-terminus of exon 9, including in-frame deletions starting at the DNA sequence corresponding to amino acids 740, 782, and 822 as well as frame-shift mutations at the DNA sequence corresponding to amino acids 761, 764, 819, and 836 (Fig. S4). Notably, mutations at amino acids 740, 782, and 836 have been suggested to generate mutant PML proteins with abnormal cytoplasmic localization (32). We speculate that these mutations observed in the clinical cases could generate exon 9 C-terminal deleted mutants with weakened control on EXO, resulting in cytoplasmic localization of PML. This aberrant localization may contribute to the process of tumorigenesis.

Although the majority of PML isoforms are nuclear, cytoplasmic PML isoforms can occur naturally. PML isoforms are generated through alternative splicing of exons 4 to 9. Exon 6, which encodes an NLS, is present in most isoforms. Exclusion of exon 6 results in cytoplasmic isoforms. One well-characterized cytoplasmic isoform is PML-VII, comprising exons 1 to 4 and a part of exon 7b without exon 6 (14, 15). This cytoplasmic PML isoform has been implicated in regulating various cellular processes, notably, the TGF-β signaling pathway. Our study suggests that excluding exon 8a from PML-I in splicing could also lead to the generation of a cytoplasmic isoform, even in the presence of the NLS. This hypothesis is supported by the identification of a murine cytoplasmic PML isoform that contains exon 9 but lacks exon 8a. This isoform is involved in regulating antigen presentation *via* MHC class I (29). We speculate that a similar PML isoform may also exist in humans, potentially regulating the normal physiological functions of cells.

## Experimental procedures

### Cell and virus

HEK293T cells were originally obtained from the American Type Culture Collection (ATCC). PML-knockout HeLa cells were previously generated in our laboratory (47). Both PML-knockout HeLa cells and HEK293T cells were cultured in Dulbecco’s Modified Eagle Medium (DMEM) (Macgene, CM10013) containing 10% fetal bovine serum (TransGen Biotech, FS301-02), 100 IU/ml penicillin and 100 μg/ml streptomycin (Macgene, CC004). The cultures were incubated in a humidified incubator containing 5% CO_2_ at 37 °C. The PRV Bartha-K61 was described previously (48).

### Plasmids construction and transfection

Plasmids expressing RFP/Flag-PML-I to -VI were previously described (47, 49). All mutants, encompassing deletions, point mutations, and fusions, were generated *via* PCR and inserted into the Flag-pSin-EF1α-puro or Prk5-HA/Flag vector. These mutants were constructed utilizing conventional molecular biological methodologies and confirmed by sequencing. A list of PCR and mutagenesis primers is shown in Table S1. Plasmids were transfected into HEK293T cells and PML-KO HeLa cells using chemifect following the manufacturer’s protocol (Fengrbio, FR-01).

Total RNA was extracted from HeLa cells using the RaPure Total RNA Kit (Magen, R4011-03) following the manufacturer’s protocol. Total RNA was reversely transcribed using M-MLV reverse transcriptase (Promega, M1708) with an oligo (dT) 18 primer to generate the cDNA library. Amplification of the TREX1 gene was carried out by PCR using the HeLa cDNA library as a template, followed by ligation into the Prk5-HA vector. Accurate sequence alignment was performed, resulting in the acquisition of the Prk5-HA-TREX1 plasmid. The mCherry-KDEL plasmid was a gift from Prof. Chen Jianguo of Peking University. The specific primers utilized in this paper are listed in Table S1.

### Lentiviral packaging and establishment of stable cell lines

HEK293T cells were transfected with Flag-pSin-EF1α-puro plasmid encoding PML-I to -VI or mutants, together with the lentiviral packaging plasmids pMD2.G and psPAX2, in a ratio of pSin/psPAX2/pMD2.G 2:2:1. After transfection for 48 h, the supernatant containing viral particles was harvested and filtered through a 0.45 μm filter. Subsequently, the filtered supernatant was utilized to infect PML-KO HeLa cells in the presence of 8 μg/ml polybrene. Cells were then subjected to treatment with 2.5 μg/ml puromycin for one week to select for cells stably expressing PML isoforms or mutants.

### Immunofluorescence

The cells were fixed with 4% paraformaldehyde for 30 min at ambient temperature and subsequently permeabilized with 0.2% Triton X-100 for 5 min on ice. After rinsing and blocking in phosphate-buffered saline (PBS) containing 1% bovine serum albumin (BSA) for 30 min, they were incubated with specific primary antibodies for 1 h at ambient temperature. After washing off the unbound antibody from cells, the cells were further exposed to the secondary antibody for 45 min. Rabbit anti-HA (1:200, Proteintech, 51064-2-AP) was used as the primary antibody, and TRITC-conjugated goat anti-rabbit antibody (1:1000, DingguoChangsheng Biotech, IF-0072) was used as the secondary antibody. Cell nuclei were stained with Dabco 4′,6-diamidino-2-phenylindole (DAPI) (Beyotime, C1005) for 3 to 5 min. Images were captured using an Eclipse Ni-E microscope (Nikon Instruments). Data acquisition was performed using a SPOT camera (Diagnostic Instruments) and NIS-Elements BR software (Nikon).

### Virus infection and titer assay

After infecting PML-KO HeLa cells with PRV viruses at 0.5 MOI for 1 h, the cells were rinsed with PBS and incubated in DMEM supplemented with 5% FBS for 24 h. Next, the supernatant was collected for viral titer assay while the remaining cells were utilized for Western blot analysis.

The viral titer in the supernatant was determined by the limited dilution method. PK15 cells were inoculated in 96-well plates at a concentration of 5 × 10^3^ cells per well, and viral detection was executed after 12 h. Supernatants collected from virus-infected PML-KO HeLa cells were centrifuged to remove cellular debris and serially diluted in serum-free DMEM at a 10-fold concentration (ranging from 10^−1^ ∼ 10^−8^) to infect PK15 cells. Cellular lesions were observed in the wells on the fourth day after inoculation, and the TCID50 was calculated.

### Immunoprecipitation and Western blot

The interaction between cytoplasmic PML-I mutants and TREX1 was examined by co-expressing Flag-PML-I-Δ(8a+9CT) mutant and HA-TREX1 in HEK293T cells, followed by the acquisition of cellular samples using lysis buffer. Subsequently, immunoprecipitation with anti-Flag M2 beads (Sigma, A2220) was performed for 4 h. The precipitated complexes were separated by SDS-PAGE and identified by western blotting.

The protein samples were lysed utilizing SDS/β-mercaptothion protein lysing buffer, followed by their separation through polyacrylamide gel electrophoresis (SDS). Subsequently, they were transferred to the PVDF membrane and detected with the indicated antibodies. Images were then exposed and captured on an ECL luminometer using the Immobilon Classico Western HRP substrate (Millipore, WBLUC0500). Primary antibodies used for analysis were anti-Flag (Sigma, M2), anti-α-Tubulin (MBL, PM054), anti-HA (Proteintech, 51064-2-AP), anti-GFP (Santa Cruz, B-2) and anti-H3 (Proteintech, 17168-1-AP). Polyclonal antibodies against PRV US3 were previously described (48).

### Nuclear-cytoplasmic fractionation assay

PML-KO HeLa cells were spread in six-well plates at 1 × 10^6^/well. After cell attachment, PML-I/-Δ9CT/-Δ(8a + 9CT) were transfected with PML-KO HeLa cells at 1 μg/well, respectively. After 24 h, cells were collected for nuclear-cytoplasmic separation using a nuclear and cytoplasmic protein extraction kit (Beyotime, P0028) following the manufacturer’s instructions. Nuclear and cytoplasmic PML proteins were extracted and detected by western blotting. Flag indicates PML proteins, Tubulin serves as a cytoplasmic control, and H3 serves as a nuclear control.

### Statistical analysis

All data were expressed as mean ± standard deviation. The open-source EzColocalization plugin for ImageJ (ImageJ Software A) was used to analyze the co-localization patterns of TREX1 and KDEL with PML mutants (50). All data were statistically analyzed using GraphPad Prism 8 software (GraphPad Software). Two-way analysis of variance (ANOVA) for at least three independent replicates based on the experimental design, and *p*-values of less than 0.05 for each test were statistically significant. ∗*p* < 0.05; ∗∗*p* < 0.01; ∗∗∗*p* < 0.001; ∗∗∗∗*p* < 0.0001; not significant, *p* > 0.05.

## Data availability

Data are contained within the article and its supporting information.

## Supporting information

This article contains supporting information.

## Conflict of interest

The authors declare that they have no conflicts of interest with the contents of this article.
