# Supplementary material for: The highly conserved region within exonuclease III-like in PML-I regulates the cytoplasmic localization of PML-NBs
Source: J Biol Chem. 2024 Oct 10;300(11):107872. doi: 10.1016/j.jbc.2024.107872 (PMC11602975; doi:10.1016/j.jbc.2024.107872)
Supplement: Supporting Information [file mmc1.docx]

**Supporting information**

**The highly conserved region within exonuclease III-like in PML-I regulates the cytoplasmic localization of PML-NBs**

Xinxin Liang, Jinwen Chen, Peijie Yan, Zhongzhou Chen, Chao Gao, Rulan Bai,

Jun Tang

**List of included material:**

**Table S1.** Primers used for mutant construction

**Figure S1.** Exon 8a deletion results in a significant reduction in the level of SUMOylation of the PML protein

**Figure S2.** Analysis of structural differences between human and spotted gar PML proteins

**Figure S3.** sgPML-exon 8a does not inhibit EXO-S

**Figure S4.** Analysis of PML-I exon 8a and exon 9 mutants in clinical samples

**Table S1. Primers used for mutant construction**

| **Primer** | **Sequence (5’-3’)** |
| --- | --- |
| ECOR I-GFP-F | ACGATGACGACAAGGAATTCATGGTGAGCAAGGGCGAGGAG |
| GFP-PML-F | ACCGCGGGCCCGGGATCCATGGAGCCTGCACCCGCCCG |
| GFP-PML-R | CGGGCGGGTGCAGGCTCCATGGATCCCGGGCCCGCGGT |
| Spe I-PML-I-R | TGCGGATCCTTCGAACTAGTTCAGCTCTGCTGGGA |
| Spe I-PML-IV-R | ATGCGGATCCTTCGAACTAGTCTAAATTAGAAAGGGGTGGGGGTA |
| Spe I -PML-VI-R | TGCGGATCCTTCGAACTAGTTCACCACAACGCGTTCCTCTC |
| PML-I-Δ8a-F | GATGCCGAAAACTCGACCCAGAAGATTAGC |
| PML-I-Δ8a-R | GCTAATCTTCTGGGTCGAGTTTTCGGCATC |
| Spe I-PML-I-Δ9-R | TGCGGATCCTTCGAACTAGTTCATTCATTGTCAATCTTGAGGTC |
| Spe I-PML-I-Δ(8a+9)-R | TGCGGATCCTTCGAACTAGTTCACGAGTTTTCGGCATCTGAGTC |
| Spe I-PML-IV-Δ8a-R | TGCGGATCCTTCGAACTAGTCTAAATTAGAAAGGGGTGGGGGTAGCCCCAGGAGAACCCACTCGAGTTTTCGGCATCTGAGTCTTCCGA |
| PML-I-ΔNES-F | CTGTGGGAATTCCAGGAGGCCATCCGGGAGCGTGTGCCCGGG |
| PML-I-ΔNES-R | CCCGGGCACACGCTCCCGGATGGCCTCCTGGAATTCCCACAG |
| PML-I-mNES-F | GAGGCCATCTCGGGCTTCGCTGCTGCCGCTCCTGCTATCCGGGAGCGTGTGCCC |
| PML-I-mNES-R | GGGCACACGCTCCCGGATAGCAGGAGCGGCAGCAGCGAAGCCCGAGATGGCCTC |
| PML-IV-9-F | CCCCACCCCTTTCTAATTACCCAGAAGATTAGCCAG |
| PML-IV-9-R | CTGGCTAATCTTCTGGGTAATTAGAAAGGGGTGGGG |
| PML-VI-9-F | GGGAGAGGAACGCGTTGTGGACCCAGAAGATTAGCCAGCT |
| PML-VI-9-R | AGCTGGCTAATCTTCTGGGTCCACAACGCGTTCCTCTCCC |
| Spe I-PML-IV-Δ8a-R | TGCGGATCCTTCGAACTAGTCTAAATTAGAAAGGGGTGGGGGTAGCCCCAGGAGAACCCACTCGAGTTTTCGGCATCTGAGTCTTCCGA |
| Δ(8a+EXO-L)-F | TCAGATGCCGAAAACTCGTCCCCGGGCCCCCAGCTG |
| Δ(8a+EXO-L)-R | CAGCTGGGGGCCCGGGGACGAGTTTTCGGCATCTGA |
| Δ(8a+EXO-S)-F | TCAGATGCCGAAAACTCGATCTCGGGCTTCCTGGCT |
| Δ(8a+EXO-S)-R | AGCCAGGAAGCCCGAGATCGAGTTTTCGGCATCTGA |
| PML-VI-EXO-S-F | GGGAGAGGAACGCGTTGTGGACCCAGAAGATTAGCCAGCT |
| PML-VI-EXO-S-R | AGCTGGCTAATCTTCTGGGTCCACAACGCGTTCCTCTCCC |
| PML-I-Δ9CT-R | TGCGGATCCTTCGAACTAGTTCAGACCTCGAGGAGGCGGCA |
| PML-VI-(8a+9)-F | GGGAGAGGAACGCGTTGTGGTCCTCCCGAGAGCTGG |
| PML-VI-(8a+9)-R | CCAGCTCTCGGGAGGACCACAACGCGTTCCTCTCCC |
| HA-TREX1-F | GACTATGCGGGCGGATCCATGGGCTCGCAGGCCCTG |
| HA-TREX1-R | GCCATGGCGGCCAAGCTTCTACTCCCCAGGTGTGGC |
| Flag-PML-F | ACGATGACGACAAGGAATTCATGGAGCCTGCACCCGCCCGA |
| Sg8a-F | AGCAGCGAGATCCAATCTAAAGTTGCCACACAAGACTGGGCAACAGGCACTATCGTGTTCTTTGATTTGGAGACCACAGGATTGGATCTCACCCAGAAGATTAGCCAGCT |
| Sg8a-R | TAGATTGGATCTCGCTGCTGATTTGCAACTGGGATACAATTTGGTTGTTATGATTCCGGCCACCTGCACCCGAGTTTTCGGCATCTGAGT |
| HA-PML1-F | GACTATGCGGGCGGATCCATGGAGCCTGCACCCGCCCGAT |
| Prk5-703-R | GGGCCATGGCGGCCAAGCTTTCAGGCCTCCTGGAATTCCCAC |

**Figure S1**


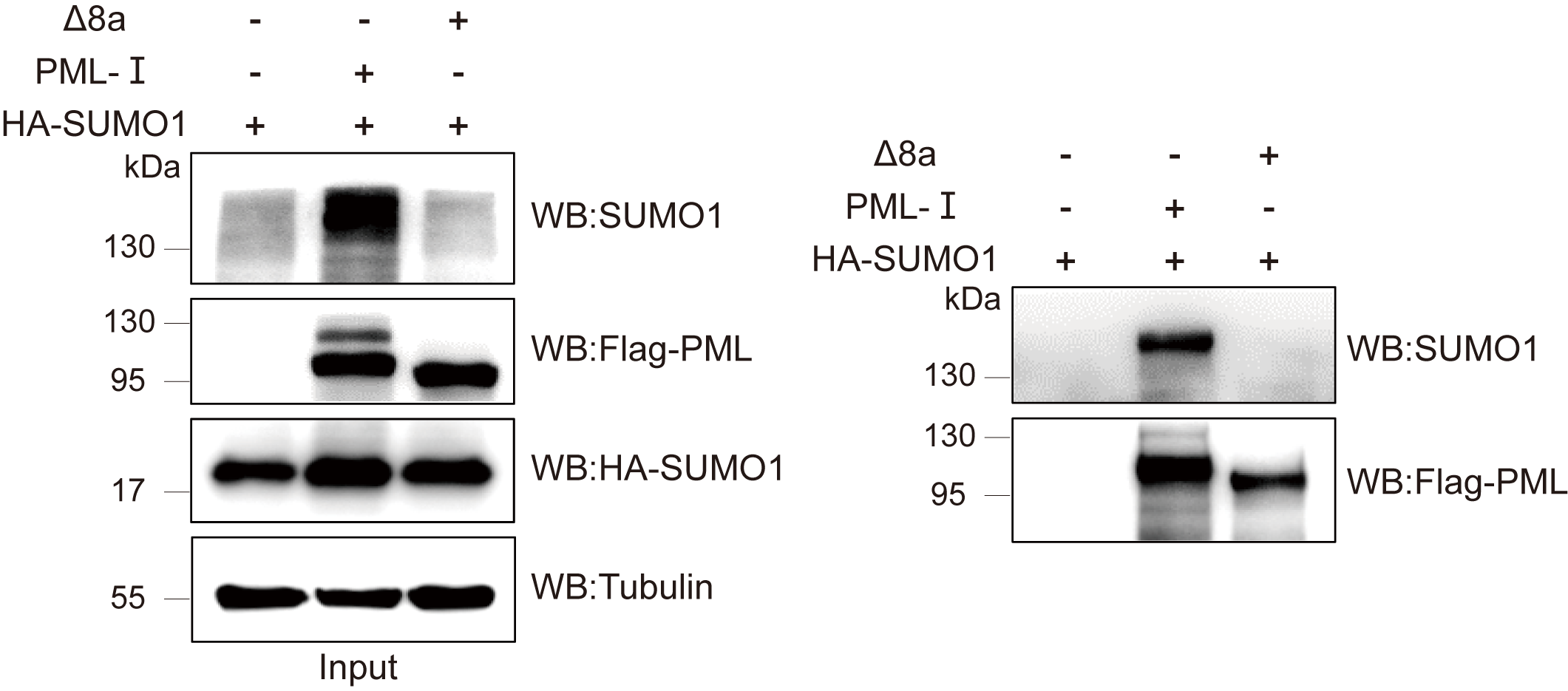


**Figure S1. Exon 8a deletion results in a significant reduction in the level of SUMOylation of the PML protein.** HEK293T cells were co-transfected with HA-SUMO-1 and Flag-PML-I or the -Δ8a mutant. Cell lysates were immunoprecipitated followed by western blot analysis using anti-HA antibodies for SUMO-1 and anti-Flag antibodies for PML.

**Figure S2**


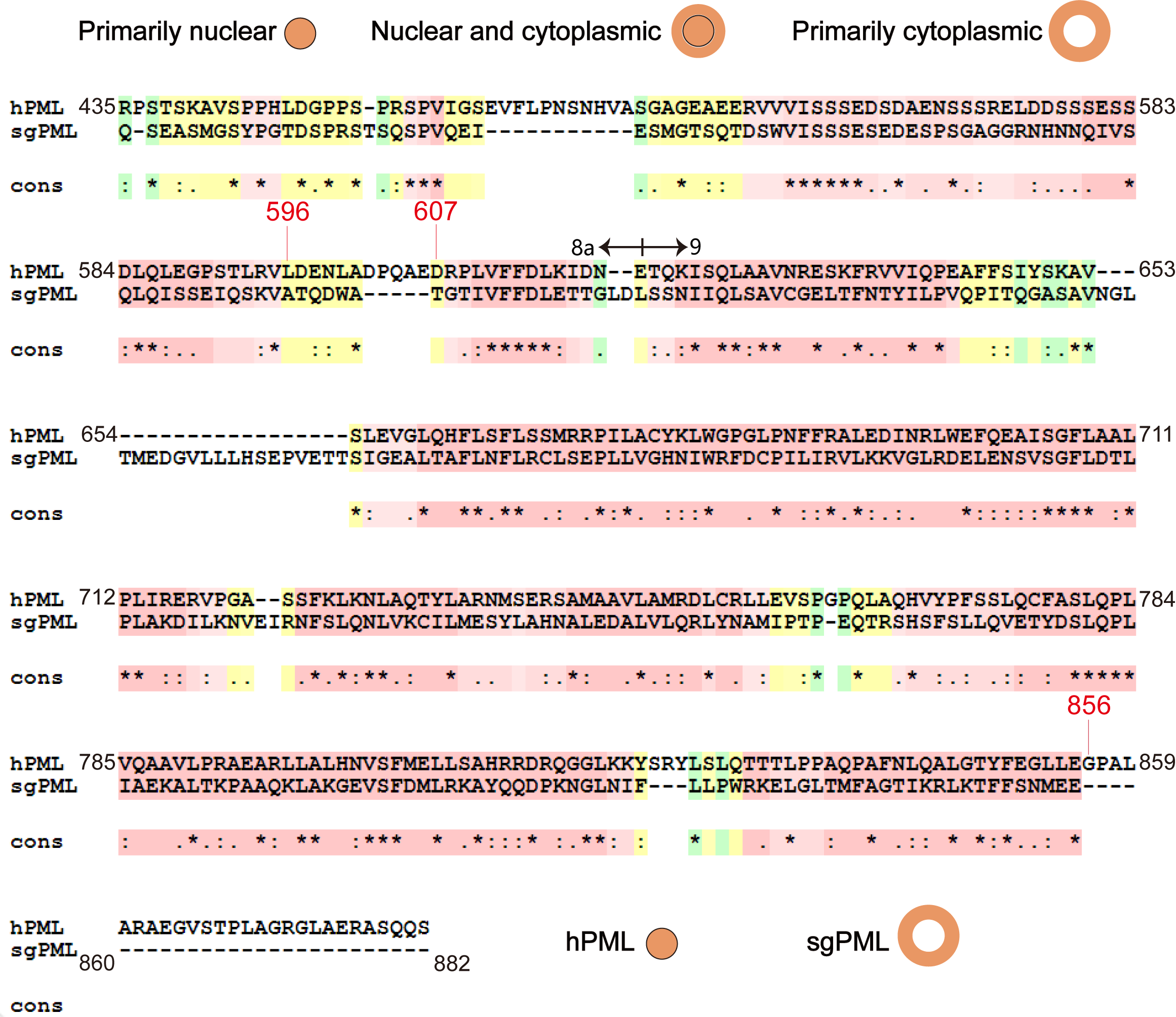


**Figure S2. Analysis of structural differences between human and spotted gar PML proteins****.** The comparison of the structural differences between human PML (hPML) and spotted gar PML (sgPML) was conducted using T-coffee (https://tcoffee.crg.eu/). The analysis includes structural information and homology extension. Regions with a higher similarity in secondary structure are highlighted in red, followed by yellow for the next best, green for the worse, and blue for the worst. Conserved amino acid sequences are marked with an asterisk (*), while two (.) indicate very similar amino acid properties, and one (.) indicates relatively similar amino acid properties.

**Figure S3**


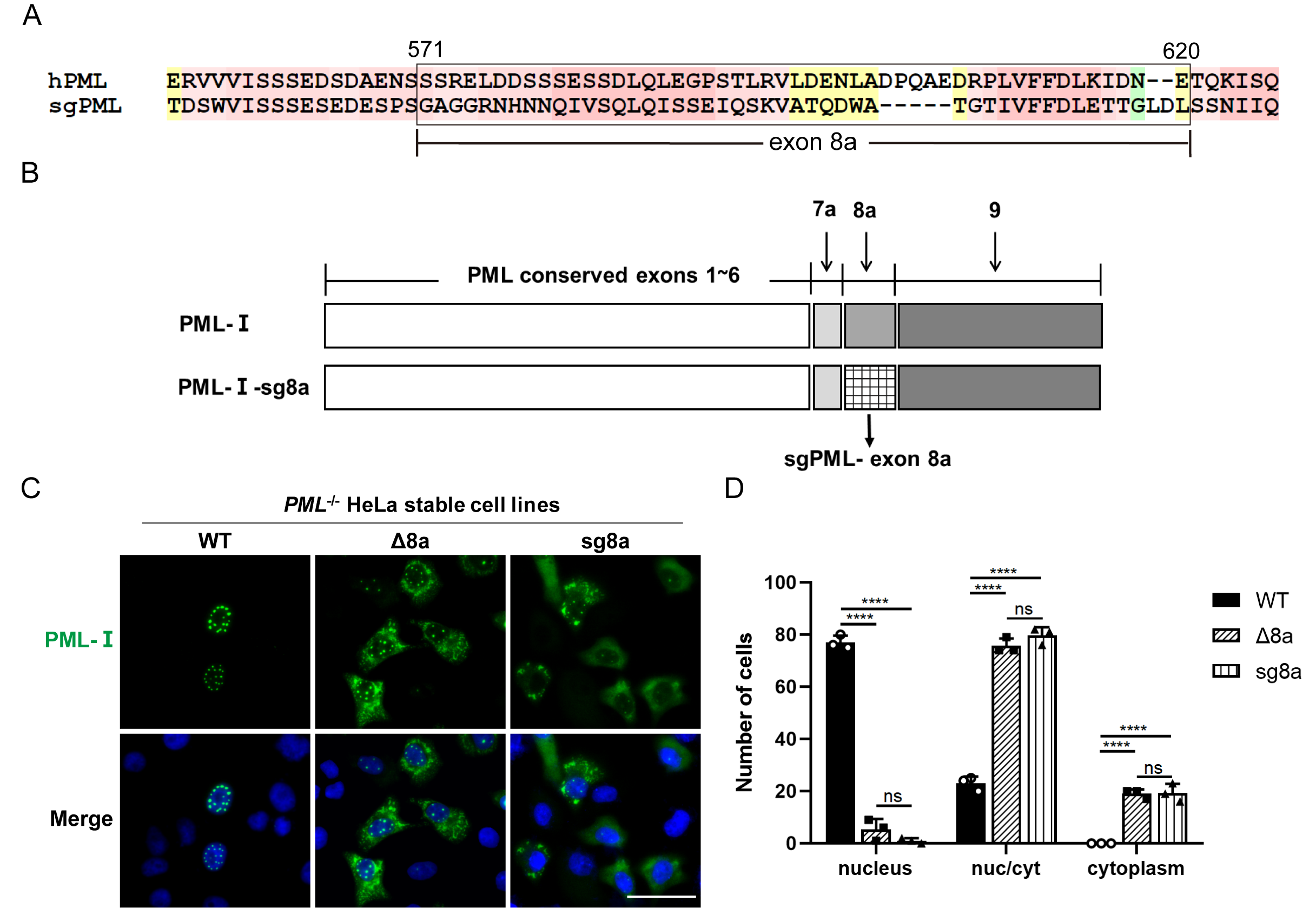


**Figure S3. sgPML-exon 8a does not inhibit EXO-S.** **(A)** Schematic representation of the replacement of exon 8a of hPML-I with exon 8a from sgPML. **(B)** Schematic depiction of the resulting PML-I-sg8a mutant, where human PML-I exon 8a was replaced with the sequence corresponding to sgPML. **(C)** Subcellular localization of the PML mutant described in (B) was observed using GFP fluorescence. Scale bar: 50 μm. **(D)** The average number of each phenotype in 100 cells from (C) was calculated and shown. Bars and error bars in (D) represent the mean with s.d. (n=3). ****P<0.0001; ns, not significant.

**Figure S4**


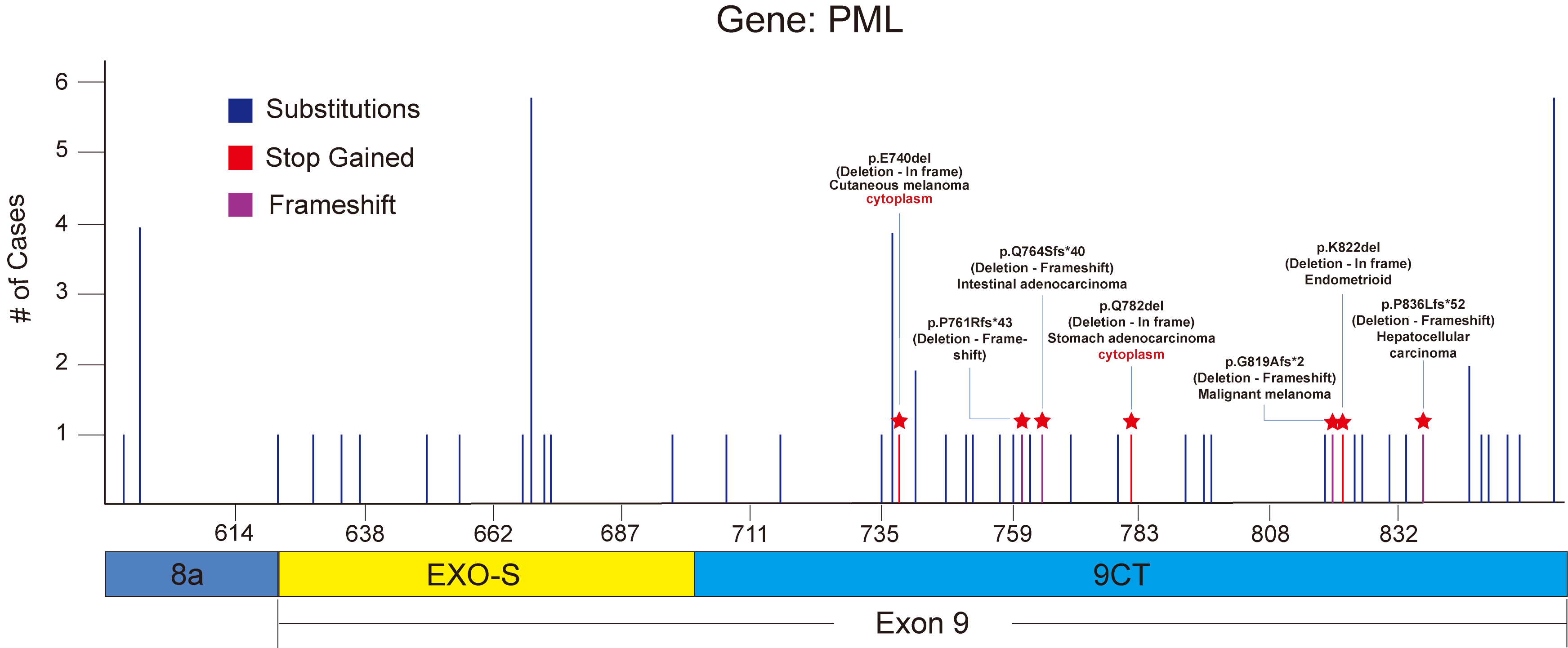


**Figure S4. Analysis of PML-I exon 8a and exon 9 mutants in clinical samples.** The result of the database search and collation of human PML-I exon 8a and 9 mutants is displayed. The vertical axis represents the number of cases, while the horizontal axis indicates the corresponding amino acid sequences on exons 8a and 9. Blue vertical lines denote substitutions, red indicates translation termination, and purple indicates code-shifting mutations. Mutations occurring at loci marked by red stars are considered high-risk. The bottom bar shows the corresponding positions on exon 8a and exon 9. Exon 8a is represented in dark blue marks, the EXO-S structural domain in yellow, and the C-terminus of exon 9 in light blue.
